# Effect of risk factors on the prevalence of influenza infections among children of slums of Dhaka city

**DOI:** 10.1186/s40064-016-2275-5

**Published:** 2016-05-11

**Authors:** Sabita Rezwana Rahman, Md Firoz Ahmed, Mohammad Ariful Islam, Md. Majibur Rahman

**Affiliations:** Department of Microbiology, University of Dhaka, Dhaka, 1000 Bangladesh; Department of Microbiology, Jahangirnagar University, Dhaka, Bangladesh; Department of Microbiology, Jagannath University, Dhaka, 1000 Bangladesh

**Keywords:** Nutrition, Influenza, Children, RT-PCR, Risk factors

## Abstract

**Background:**

Influenza viruses may cause severe acute respiratory illness among human population. People of densely populated areas, e.g., slum, are mostly affected by influenza viruses. Although potential vaccines to influenza viruses have been developed, infection rate is still high, therefore, increase the morbidity and mortality rate in slum areas. To treat these infections, slum dwellers including children and mothers do not get proper medication as well as vaccination. Hence, prevention remains to be the only mean to tackle such infections. Herein, we determined the prevalence of influenza infections among nutritionally deprived children and mothers of slum areas in Dhaka city and demonstrated the association with different risk factors like age, gender and socio-economic status.

**Results:**

Nasopharyngeal swab samples and a short demography of all the participants suffering from influenza-like illness (ILI) were collected. The samples were subjected to RNA extraction and then real-time RT-PCR to detect influenza viruses. Among the ILI patients, about 87.9 % did not have knowledge about influenza infections and 80.5 % did not cover their noses during coughing as well as sneezing. Children were significantly infected by both influenza A and influenza B viruses, suggesting their vulnerability to these infections. Additionally, among the children with ILI, influenza infections were significantly associated with age below or equal to three years, very poor family incomes, practicing unhygienic habits and nutritional deficiency.

**Conclusions:**

This study suggests that proper vaccination, improved sanitary conditions and nutritional diet may help reduce the risk of influenza infections in slum areas.

## Background

Influenza viruses cause a large portion of respiratory illness which sometimes reach epidemic level of infection (Belshe et al. 2007; Yusuf et al. 2007; Abdullah Brooks et al. 2007). Seasonal respiratory infection is an important cause of hospitalization and excess mortality not only in winter season but also all year-round (Ou et al. 2013; Weerasinghe et al. 2002; Woodhouse et al. 1994; Koul et al. 2014). Globally, more than 1 billion people are infected annually by influenza viruses, whereas, most of them are from developing countries including Bangladesh (Kamlangdee et al. 2014; Sydnor and Perl 2011; Fedson 2009; Oshitani et al. 2008). Studies of influenza infections documented that in Bangladesh more than 50 % of the child respiratory infections are caused by influenza viruses (Nasreen et al. 2014; Bhuiyan et al. 2014).

Influenza virus is a negative-stranded RNA virus whose genome consists of eight segments encoding at least 10 polypeptides (Elton et al. 2001; Obayashi et al. 2008). The influenza viruses are divided into several types based on the antigenic differences of nucleoprotein and matrix proteins. Additionally, genome reassortments and emerging mutations sometime spawn new strains of influenza viruses as imminent epidemic or pandemic threats to human health (Mehle et al. 2012). Among different types of influenza viruses, type A is the most severe which further can be subtyped on the basis of antigenic differences of external glycoproteins, hemagglutinin (HA) and neuraminidase (NA) (Wright et al. 1995; Shaha et al. 2015). On the other hand, influenza B viruses are relatively less severe and develop infections among children (Abdullah Brooks et al. 2007; Zaman et al. 2009).

Slums with heavily dense population are one of the main reservoirs of influenza infections (Streatfield and Karar 2008). Previous study documented that Bangladesh has more than 9081 slums with very poor sanitation system and lowest economic condition (Islam et al. 2006). Different diseases appear all year-round in these areas with the increased morbidity and mortality rates (Khatun et al. 2012; Vaid et al. 2007). Many studies were performed about the prevalence of influenza infections in urban slums but the actual reasons for such diseases were poorly understood (Streatfield and Karar 2008; Abdullah Brooks et al. 2007; Rashid 2009).

In this study, we demonstrated the presumptive causes of influenza like illness (ILI) as well as the prevalence of influenza infections among mothers and children in slums of Dhaka city. We also tried to determine the association of influenza infections with different risk factors like age, gender, socio-economic status (annual income) and occupation of mothers.

## Results

The slums of Dhaka city are such densely populated that it invites transmission of influenza from person to person leading to a probable complication of primary viral pneumonia and often secondary bacterial pneumonia which eventually could lead to death. Although potential vaccines have been developed the slum dwellers have a little access to those. Hence, prevention remains to be the only means of controlling such life threatening diseases. In this study we have determined the prevalence of influenza infections among nutritionally deprived children and mother of slum areas of Dhaka, Bangladesh.

### Analysis of physical conditions related to influenza like illness

Out of 540 participants with ILI in urban slums, 200 participants were mothers and 340 participants were children (male: 160 participants; female: 180 participants). The mean ages of the mothers and children were 31 and 4 years respectively. The occupations of participated mothers were most likely to be domestic workers, day labors, garment workers and housewives. The annual family incomes of all participants varied from US$ 800 to US$ 1500. About 87.9 % of the participants did not have knowledge about influenza infections and 80.5 % of the participants did not cover their noses during coughing and sneezing. Besides the availability of influenza vaccines, none of them were immunized which might be because of their lack of awareness. Additionally, almost 45.6 % of the participants had regular exposure to pet animals. Among the mothers, the percentages of washing hands before eating and after using toilet were 55.7 and 67.8 % (data not shown) respectively.

### Association of influenza like illness (ILI) with different socio-demographic characteristics

**Fig. 1:**
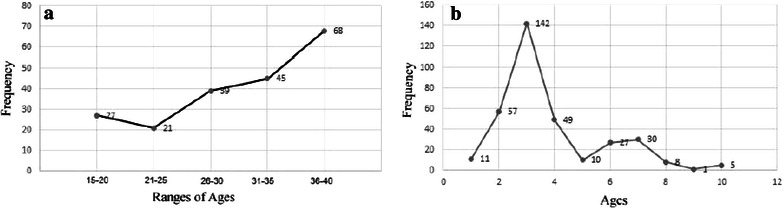
Relation of influenza like illness with ages of the respondents. **a** Age ranges of mothers; **b** Ages of children

**Fig. 2:**
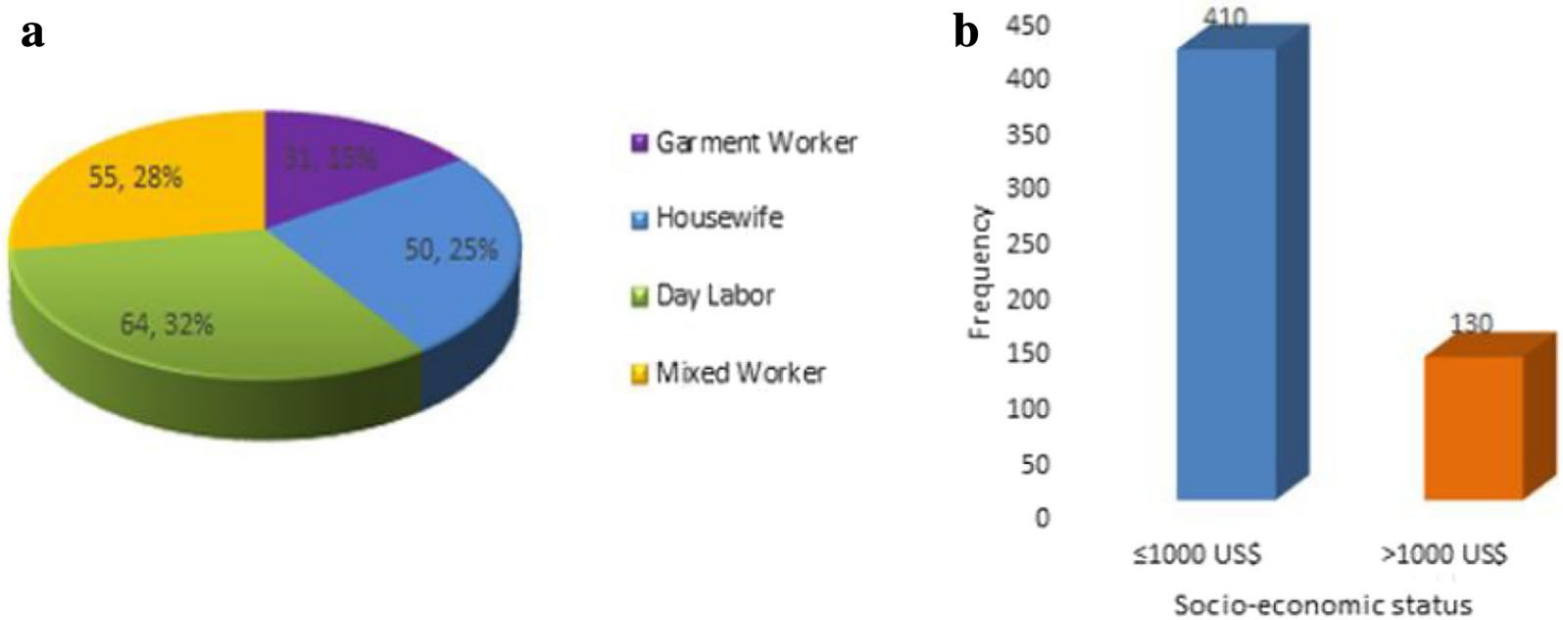
Association of influenza like illness with mothers’ occupation and family socio-economic status. **a** Mothers occupation; **b** Family socio-economic status

The occurrence of ILI was observed high among the mothers of 36–40 years age range, while the mothers of 21–25 years age range were least affected (Fig. 1a). On the other hand, the number of children of 3 years were significantly prone to be affected by ILI which tend to decrease gradually with increasing age (Fig. 1b). Approximately, 64 % of the mothers with ILI were day laborer (Fig. 2a). With regards to the socio-economic status, 76 % of the participants were with very low income, less than 1000 US$ per annum (Fig. 2b).

### Detection of influenza viruses by rRT- PCR

Among 540 samples, 110 (20.4 %) samples tested positive for influenza viruses by rRT-PCR. Of that, 61 (55.5 %) samples (mothers: 11 and children: 50) were positive for influenza A virus and 49 (44.5 %) samples (mothers: 11 and children: 38) were positive for influenza B virus. Children were significantly infected by both influenza A (POR 2.96, 95 % CI 1.5038–5.8355, P: 0.002) and influenza B viruses (POR 2.16, 95 % CI 1.0787–4.3333, P: 0.03) as shown in Table 1a and b respectively. Additionally, among 340 ILI positive children, influenza infections had significant negative association with the increasing ages of children (POR 1.94, 95 % CI 1.1377–3.2947, P 0.01) and annual family incomes (POR: 2.05, 95 % CI 1.1361–3.7034, P 0.02) (Table 2). Of the influenza A virus positive samples 25 % were subtyped as H3N2 and 15 % were subtyped as H1N1.

**Table 1:** Prevalence of influenza A and B infection among ILI mother and children

Groups	IAV positive	IAV negative	POR (95 % CI)	*P* value
Prevalence of influenza A infection among ILI mother and children				
Children (n = 340)	50	290	2.96 (1.5038–5.8355)*	0.002*
Mothers (n = 200)	11	189		
Prevalence of influenza B infection among ILI mother and children				
Children (n = 340)	38	302	2.16 (1.0787–4.3333)*	0.03*
Mothers (n = 200)	11	189		

*IAV* influenza A virus, *IBV* influenza B virus, *POR* prevalence odds ratio, *CI* confidence interval, *P* provability

*Denotes statistically significant

**Table 2:** Prevalence of Influenza infections among ILI positive children according to socio-demographic characteristics

Factors		Influenza positive	Influenza negative	POR (95 % CI)	P value
Age (years)	≤03 (210)	64	146	1.94 (1.1377–3.2947)*	0.01*
	>03 (130)	24	106		
Gender	Male (160)	40	120	0.79 (0.4851–1.2727)	0.33
	Female (180)	48	132		
Socio-economic status (SES)^a^	≤1000 US$ (240)	71	169	2.05 (1.1361–3.7034)*	0.02*
	>1000 US$ (100)	17	83		

*POR* prevalence odds ratio, *CI* confidence interval, *P* provability

* Denotes statistically significant

^a^Denotes annual family income of the respective families

## Discussion

To our knowledge there is no slum population based study to evaluate and compare occurrence of ILI in slum dwellers of different age group, sex and economic status. The burden of influenza associated illness was found to be dependent on hygiene condition. Most slums were damp and had open sewage connection even inside the temporary houses built with straw and bamboo.

Similar to the observations in the present study, numerous studies in other contexts also found a comprehensive relationship of getting ILI with very low income, lack of hand washing, ignorance about influenza infections, and dirty environmental conditions (Morens and Taubenberger 2010; Ram et al. 2015). However, the underlying reasons are poorly understood. The objective of this study was to determine whether there is any risk factor that modulates the prevalence of influenza infections among children of slum areas.

The occurrence of ILI was observed high among the mothers of 36–40 years age range and children of 3 years, which is consistent with other study (Iskander et al. 2009). One plausible reason behind it might be their compromised immune system as most of the participants were from very poor family and may not provide adequate nutrition required to maintain a sound health (Fig. 2) (Albers et al. 2013).

Real time RT-PCR is one of the latest techniques to detect viral gene directly from the samples using specified probes. In this study, we used this potential technique to identify influenza A and B viruses from slum dwellers. Among the ILI positive patients, 110 (20.4 %) patients tested positive for influenza viruses, of which approximately 55.5 and 44.5 % were specifically positive for influenza A and B viruses respectively. As we observed that children were three times more infected by influenza A viruses and two times more infected by influenza B viruses than mothers (Table 1). Besides being having compromised immune system children residing in slum are unattended as their parents are part time workers in different households. Children frequently visit the unhygienic areas of slums knowingly and/or unknowingly, this is because a child of 3 years is not so concerned about hygiene. On the other hand, getting unexpected exposure to infectious viruses may also be a vital cause of infection. This finding was in agreement with that of Paulo et al. (2010). A similar finding was also reported in rural Kenya where older sensible children were less likely to be infected (Breiman et al. 2015).

With regards to the socio-demographic characteristics, the prevalence of influenza infections is significantly higher in less than or equal of 3 years of age of children and very poor families of the slum areas. Studies in other contexts of the world have also documented similar findings (Yang et al. 2015; Iwasaki and Pillai 2014) but the actual fact has remained unclear. One plausible explanation is that the children are not conscious about personal hygiene and the sanitation systems of that areas as stated above. Other studies have also found that influenza viruses get a very favorable opportunity to infect a wide range of susceptible individuals in the slum area because of poor sanitation systems (Verreault et al. 2008). Furthermore, very poor income families might suffer from nutritional deficiency which is one of the key factors to combat influenza infections (Iskander et al. 2009). The findings of this study suggested that children were more vulnerable to influenza infections than mothers, although other risk factors were observed to uphold the infections. Therefore, the association between influenza infections and other immunomodulating risk factors needs to be demonstrated.

## Conclusions

Being severely infectious agent, influenza viruses have a very high prevalence in the slum areas and children are the mostly affected. This study is important for two potential reasons. First, measuring the ILI status along with influenza viruses among poor slum dwellers who are deprived of the basic needs to live soundly. Second, importantly, children of 3 years of age were highly infected with influenza A and B viruses that might cause a high mortality rate and thus, the country may loss a large portion of young and healthy manpower in the near future. This study alerts public health authorities in Bangladesh about the need to upgrade living conditions and healthcare in the slum areas.

## Methods

### Sample collection and study population

Nasopharyngeal swab specimens were collected by trained laboratory technicians from 540 patients with influenza like illness who were nutritionally deprived and reported to physician during September–February, 2015 from different slums in Dhaka city with their written consent. All participants of this study had ILI with symptoms like cough, fever, running nose, sore throat, and/or headache. The study was approved by the ethical committee of Dhaka Medical College, Dhaka-1000, Bangladesh (reference number: DMC-MEU/ECC/2014/17). All the patients participated in this study were asked to fill a questionnaire, which includes the ages, socio-economic status, occupation, practice of hygienic condition, nutrition status of their family along with the environmental condition. For the collection of samples of nasal swab, a dry cotton tipped swab was inserted into the nostril parallel to the palate and left in place for a few seconds before being slowly withdrawn using a rotating motion. Specimens from both nostrils were obtained with the same swab. The tip of the swab was placed into a collection vial containing 2 ml of viral transport medium (VTM) and the applicator stick was then broken off. Samples were then placed at 4 °C immediately after collection and promptly transported to the laboratory.

### Extraction of viral RNA and real-time reverse transcriptase polymerase chain reaction (rRT-PCR)

Viral RNA was extracted from the collected samples using STRATEC Molecular RTP^®^ Pathogen Kit (Berlin, Germany) according to the manufacturer’s instructions. The extraction was carried out in a Bio-Safety Level-II (BSL-II) cabinet. The viral nucleic acid was stored in aliquots at −80 °C until use. Quantitative PCR (qPCR) was performed using a one-step RT-PCR kit (Invitrogen, USA) with a 25 µl reaction mixture containing 5 μl of RNA template, 12.5 µl 2× buffer, 1 µl enhancer, 1 µl 50× taq polymerase enzyme, 0.5 µl 100× forward primer, 0.5 µl 100× reverse primer, 0.5 µl 100× probe, 4 µl nuclease free water. The PCR cycling conditions consisted of an initial reverse transcriptase step at 50 °C for 30 min, followed by a 5 min hold at 95 °C, and then 45 cycles of 15 s at 95 °C and 30 s at 55 °C. Results were documented considering the relative cycle threshold (Ct) values with that of positive control. High Ct values (>38) were avoided. The primer sequences used for influenza A: Forward, GAC CRA TCC TGT CAC CTC TGA C; Reverse, AGG GCA TTY TGG ACA AAK CGT CTA; Probe, TGC AGT CCT CGC TCA CTG GGC ACG; Influenza B: Forward, TCC TCA AYT CAC TCT TCG AGC G; Reverse, CGG TGC TCT TGA CCA AAT TGG; Probe, CCA ATT CGA GCA GCT GAA ACT GCG GTG; RnaseP: Forward, AGA TTT GGA CCT GCG AGC G; Reverse, GAG CGG CTG TCT CCA CAA GT; Probe, TTC TGA CCT GAA GGC TCT GCG CG.

### Statistical analysis of the obtained data

Data were analyzed with MedCalc v11.3.0.0; the association of influenza infections with risk factors was analyzed by estimating prevalence odds ratios (POR). Statistical significance was assessed by calculating 95 % confidence interval (CI). Chi square test was also performed to analyze the level of significance (P < 0.05).

## Abbreviations

SES: socio-economic status
ILI: influenza like illness
rRT-PCR: real-time reverse transcriptase PCR
IAV: influenza A virus
IBV: influenza B virus
HA: hemagglutinin
NA: neuraminidase
VTM: viral transport medium
POR: prevalence odds ratio
CI: confidence interval
